# Multi-year analysis of the global preclinical antibacterial pipeline: trends and gaps

**DOI:** 10.1128/aac.00535-24

**Published:** 2024-07-15

**Authors:** Valeria Gigante, Richard A. Alm, Daniela Melchiorri, Tamarie Rocke, Cesar A. Arias, Lloyd Czaplewski, Prabhavathi Fernandes, François Franceschi, Stephan Harbarth, Roman Kozlov, Christian Lienhardt, Norio Ohmagari, Lesley A. Ogilvie, Mical Paul, John H. Rex, Lynn L. Silver, Melvin Spigelman, Hatim Sati, Alexandra M. Cameron

**Affiliations:** 1 AMR Division, World Health Organization, Geneva, Switzerland; 2 Combating Antibiotic Resistant Bacteria Biopharmaceutical Accelerator, Boston, Massachusetts, USA; 3 WHO Consultants to the WHO AMR Division, Geneva, Switzerland; 4 Department of Physiology and Pharmacology, Sapienza University, Rome, Italy; 5 Center for Infectious Diseases Research, Houston Methodist Research Institute, Houston, Texas, USA; 6 Chemical Biology Ventures Ltd., Abingdon, United Kingdom; 7 National Biodefense Science Board, U.S. Department of Health and Human Services, Washington, DC, USA; 8 Global Antibiotic Research & Development Partnership, Geneva, Switzerland; 9 Infection Control Programme, Geneva University Hospitals, Geneva, Switzerland; 10 Faculty of Medicine, WHO Collaborating Center for Patient Safety, Geneva, Switzerland; 11 Institute of Antimicrobial Chemotherapy, Smolensk State Medical University, Smolensk, Russia; 12 Université de Montpellier, INSERM, Institut de Recherche pour le Développement, Montpellier, France; 13 National Center for Global Health and Medicine, Tokyo, Japan; 14 Global Antimicrobial Resistance Research and Development Hub, Berlin, Germany; 15 Infectious Diseases, Rambam Health Care Campus, Haifa, Israel; 16 The Ruth and Bruce Rappaport Faculty of Medicine, Technion Israel Institute of Technology, Haifa, Israel; 17 F2G, Limited, Eccles, United Kingdom; 18 AMR Solutions, Boston, Massachusetts, USA; 19 Advent Life Sciences, London, United Kingdom; 20 McGovern Medical School, The University of Health Science Center at Houston, Houston, Texas, USA; 21 LL Silver Consulting, Springfield, New Jersey, USA; 22 Global Alliance for TB Drug Development, New York, New York, USA; Tufts University - New England Medical Center, Boston, Massachusetts, USA

**Keywords:** antibiotic, antimicrobial resistance, pipeline, drug discovery

## Abstract

Antimicrobial resistance (AMR) is a major global health threat estimated to have caused the deaths of 1.27 million people in 2019, which is more than HIV/AIDS and malaria deaths combined. AMR also has significant consequences on the global economy. If not properly addressed, AMR could immensely impact the world’s economy, further increasing the poverty burden in low- and middle-income countries. To mitigate the risk of a post-antibiotic society, where the ability to effectively treat common bacterial infections is being severely threatened, it is necessary to establish a continuous supply of new and novel antibacterial medicines. However, there are gaps in the current pipeline that will prove difficult to address, given the time required to develop new agents. To understand the status of upstream antibiotic development and the challenges faced by drug developers in the early development stage, the World Health Organization has regularly assessed the preclinical and clinical antibacterial development pipeline. The review identifies potential new classes of antibiotics or novel mechanisms of action that can better address resistant bacterial strains. This proactive approach is necessary to stay ahead of evolving resistance patterns and to support the availability of effective treatment options. This review examines the trends in preclinical development and attempts to identify gaps and potential opportunities to overcome the numerous hurdles in the early stages of the antibacterial research and development space.

## INTRODUCTION

The global mortality burden directly attributable to bacterial antimicrobial resistance (AMR) has been recently estimated at 1.27 million people in 2019 (95% confidence interval [CI]: 0.91 to 1.71 million), with disproportionate impact on both the very young as well as those living in lower-resource settings (1). AMR also jeopardizes the global economy and could cost up to USD 100 trillion by 2050.

This impact is projected to mostly be shouldered by low- and middle-income countries, dragging an additional 28 million people into poverty (2). Despite a plateau in overall AMR burden within the next 10–20 years in most countries, there will be a twofold increase in resistance to reserve antibiotics (3). The World Health Organization (WHO) has deemed AMR as one of the top 10 threats to global health, for which the lack of a suitably sustainable development pipeline is a key challenge (4).

Since the introduction of the first antibiotic into mainstream clinical practice in the 1940s, antibiotics have been considered the foundation of modern medicine. Many life-saving medical advances would not have been possible without the reliable action of antibiotics. However, while there was a rich pipeline of new agents developed in the middle of the 20th century with several agents being granted marketing authorization, the rate of new approvals for antibacterial agents has dramatically decreased. Over the last 12 years, the average number of approvals has been 1.2 agents per year globally (5). The high risk of development failure and insufficient return on investment for commercialized products are contributing factors, the latter being due to the frequent classification of new products as last-resort options for multi-drug-resistant infections. There is an urgent need for solutions to replenish the depleted antibacterial pipeline and to establish sustainable reimbursement pathways for product commercialization, ensuring long-term viability of research and development (R&D) and ultimately access. Furthermore, the inequitable access to quality antibiotics, including newly approved reserve antibiotics to treat infections globally, further exacerbates the burden associated with AMR development (2, 6, 7).

The WHO released the first Bacterial Priority Pathogens List (BPPL) in 2017 (8, 9) with the aim to guide R&D of new antibacterial agents, vaccines, and diagnostics against resistant bacterial species with the greatest unmet medical need (5, 6). Two years after the release of the first BPPL in 2017, the first preclinical pipeline review was performed and was used to gauge how the global research ecosystem had responded since the WHO global priority-setting exercise. Since 2019, WHO reviews the preclinical pipeline on an annual basis to monitor the preparedness of the antibacterial R&D ecosystem in responding to the priority pathogens identified through the BPPL. The most recent antibacterial clinical pipeline review (5) confirms that very few agents in clinical development effectively target the critical Gram-negative bacteria, considered to be the highest priority for R&D, and only a small number of the products assessed meet WHO’s innovation criteria. While the outlook appears positive for contemporary approaches to discover new drugs, there has been limited success in finding clinical candidates particularly for activity against Gram-negative bacteria (10, 11). Understanding the innovation potential of products under development is a critical step for new agents as these are the only antibacterials that have lower chances of cross-resistance to existing antibiotics. To complement WHO’s analysis of antibacterial products in clinical development, WHO has also undertaken a regular review of the preclinical pipeline since 2019 (12). The preclinical review aims to provide a snapshot of the early development stage and to identify innovative products that may move forward to the clinical pipeline and eventually reach the market and patients. These reviews assist in identifying trends in the broader antibacterial R&D ecosystem. However, fully analyzing the preclinical pipeline poses a challenge as not all programs are disclosed and because of a higher turnover rate as compared to the clinical pipeline due to lack of scientific progress and resource availability. Greater transparency in both the preclinical and clinical pipelines can lead to stronger collaboration around potentially innovative but challenging projects, support a community of scientists and drug developers, and generate more interest and funding into drug development for novel antibacterial agents. This article provides an analysis of the 2023 preclinical pipeline and provides a multi-year analysis of trends in the preclinical pipeline between 2019 and 2023.

## METHODOLOGY

### Scope and inclusion/exclusion criteria

This preclinical pipeline analysis focuses on antibacterial agents that target the 2024 WHO priority pathogens (13) and *Clostridioides difficile* that are in lead optimization (post-hit expansion) through to the filing of an investigational new drug (IND) application or a clinical trial application to initiate human testing. The scope includes both traditional and non-traditional programs. Traditional agents are usually small molecules, direct-acting agents that kill bacteria or inhibit their proliferation. Non-traditional agents cover anything that is different from a direct-acting small molecule such as bacteriophages, antibodies, lysins, live biotherapeutics, oligonucleotides, peptides, antivirulence agents and biofilm disruptors, potentiators, microbiome modifying agents, and immunomodulators. The review includes also repurposed non-antibiotics, antibiotics used in animals being repurposed to human use, de-colonization agents, and combination therapies. The analysis does not include vaccines, diagnostics, antifungals, antivirals, or antiparasitics. Wound care agents, non-specific supportive treatments, medical devices, and industrial or veterinary agents are also not included.

### Search strategy

The latest primary data were collected through an online data call published on the WHO web page during the first half of 2023. These data were supplemented with information from the Beam Alliance (https://beam-alliance.eu/), CARB-X (https://carb-x.org/), Novo Repair Impact Fund (https://www.repair-impact-fund.com/), and INCATE (https://www.incate.net/). In addition, programs from earlier years were checked through a desk review and, where required, updates were solicited by email. The data presented were self-declared from the institutions, and where possible, WHO confirmed the integrity through publications, conference abstracts or posters, institutional websites, direct contact, and other information in the public domain.

### Approach

Clinical and preclinical programs were analyzed separately. The overall numbers, the turnover, and attrition of preclinical programs were reviewed across the 4 years of pipeline analyses (2019–2023). In addition, the most frequent type of entities encountered (academic, commercial, non-profit), size and ownership (i.e., private versus public companies), and their geographical distribution were also examined across the 2019–2023 time frame. In 2022, no data were collected; the 2021 WHO pipeline analysis was based on data collected in 2021 and published in 2022.

## ANALYSIS AND DISCUSSION

### The number of preclinical antibacterial research programs and research groups is tiny

**Fig 1:**
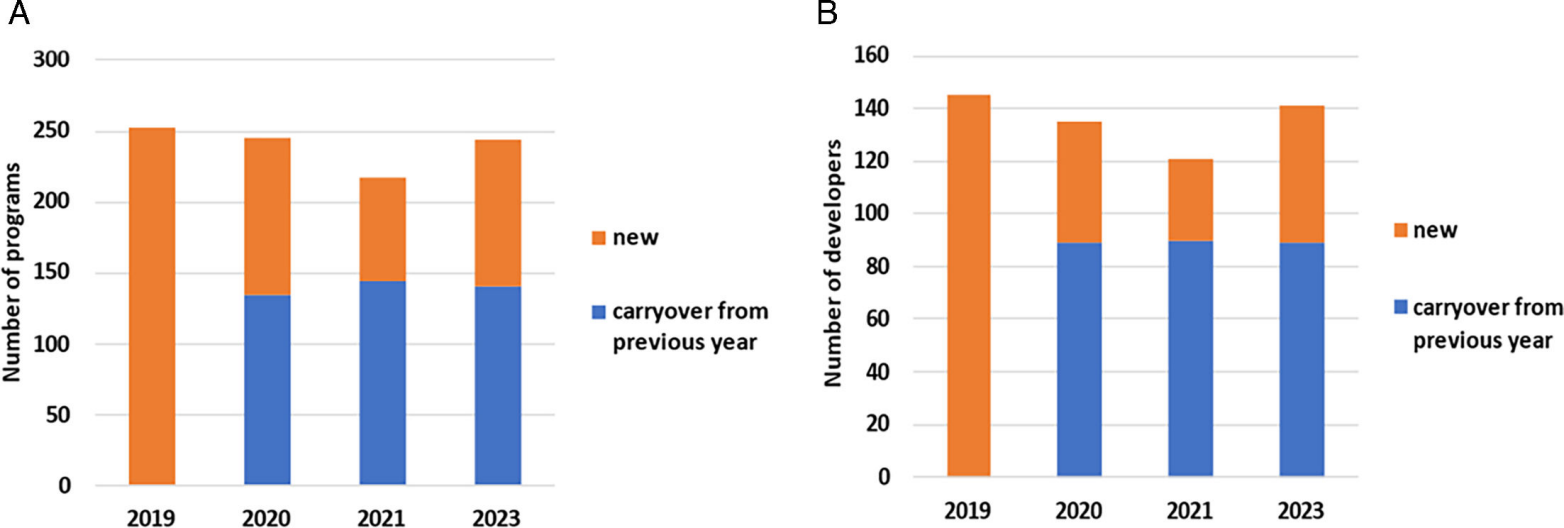
Total number of preclinical candidates (**A**) and developers (**B**) across the four pipeline analyses performed since 2019. 2022 does not appear as the 2021 review was based on 2021 data and was published in 2022. Note: the preclinical pipeline developers include academic institutions, companies, or foundations.

The latest preclinical pipeline analysis identified a total of 244 potential candidates conducted by 141 research groups of developers (note that this includes academic, commercial, and non-profit groups and private or public companies). A project is typically declared a candidate after the lead optimization phase of drug development. This figure remains consistent with previous years, maintaining a range between 217 and 252 candidates and between 121 and 145 developers (Fig. 1). However, only 30% of lead compounds tested in the preclinical setting will potentially enter clinical trials (14). The number of researchers in the AMR area overall is also small, estimated to be around 3,000 researchers, which is approximately 10 times fewer than those involved in oncology research (15).

**Fig 2:**
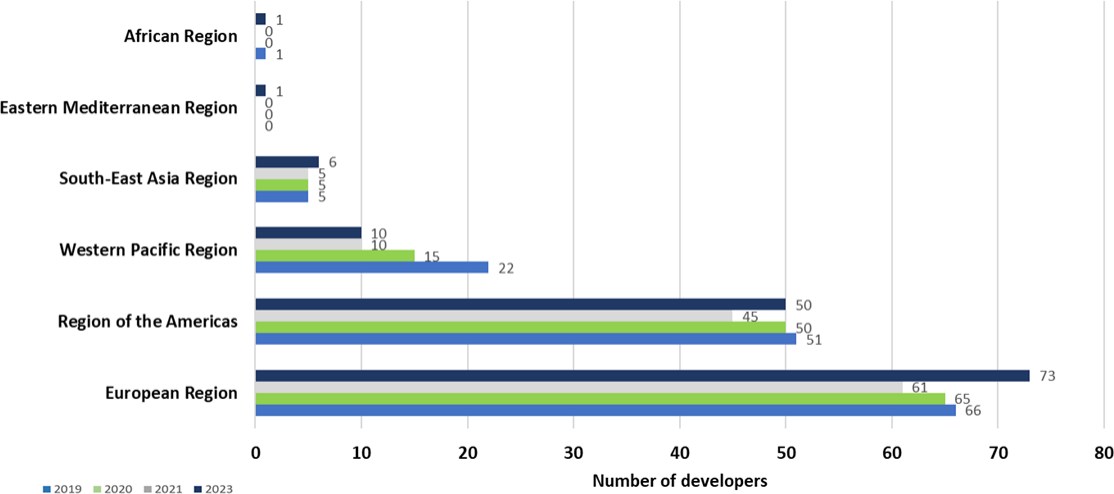
Geographical distribution of the developers with preclinical pipeline projects across the 2019–2023 analysis.

From those answering the WHO data call, most of the research groups developing novel therapeutics were in Europe (range of 45.5%–51.8% of the submissions) and the Americas (range of 35.2%–37.2% of the submissions), mostly from North America. A plausible explanation for this is that most funding opportunities likely come from these geographies where experienced researchers with R&D know-how have been trained and settled over several decades. A breakdown of the location of the unique research groups is provided in Fig. 2. In addition, the WHO will make publicly available all data on its website through The Global Observatory on Health R&D (https://www.who.int/observatories/global-observatory-on-health-research-and-development/monitoring/who-antibacterial-preclinical-pipeline-review).

Importantly, there was a high turnover of active preclinical programs. Out of 269 unique developers included in the analyses, only 51 groups (19%) have remained consistently active across all four survey years from 2019 to 2023. Consistent with the high failure rate of early pharmaceutical R&D in general (16), this turnover suggests a substantial attrition rate (note that the term “attrition rate” in this context refers to the rate at which programs leave or drop out of all preclinical programs tallied over a specified period, expressed as a percentage of the total number of programs tallied at the beginning of that period), with estimates indicating that between 45% and 60% of the preclinical ecosystem has been lost over the time frame of these reports. The high turnover in the preclinical development of antibacterial agents can be attributed to several factors related to safety, quality, and financial considerations. Key issues include unacceptable toxicity in animal models, manufacturing problems, and challenges related to the chemical or biological properties of the compound. Additionally, business considerations, such as the lack of profitability or insufficient funding, can also lead to a decision to halt development.

**Fig 3:**
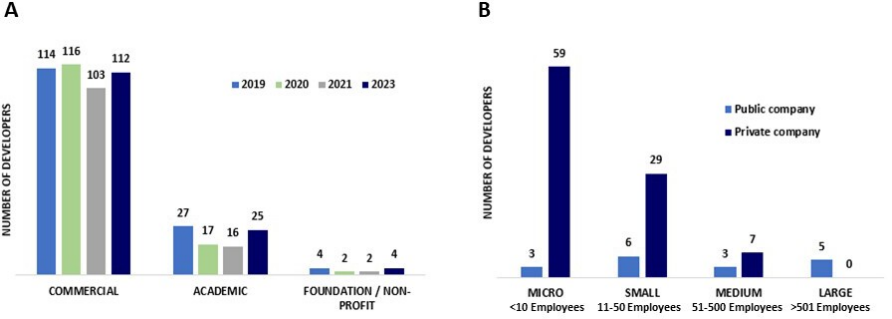
Categorization of developers with preclinical pipeline projects by type (**A**) and by ownership and size for the commercial companies in 2023 (**B**).

The majority (between 78.6% and 85.9%) were commercial entities, while the remainder were academic institutions and non-profit organizations (Fig. 3A). When commercial entities were further stratified based on their funding source, over 80% of the commercial entities were privately funded (Fig. 3B). Additionally, a consistent trend across the years of the analysis shows that about 50% of these companies were defined as “micro” enterprises (having less than 10 employees), and another 25%–30% had only a slightly larger workforce (Fig. 3B). The paucity of small, privately funded commercial entities engaged in preclinical antibacterial research underscores the fragility of the preclinical ecosystem and the future pipeline.

The small number of entities engaged in preclinical antibacterial research parallels the scarcity of large pharmaceutical companies in this field since a notable departure of significant pharmaceutical players from antibacterial discovery. However, it should be noted that the few large pharmaceutical companies actively involved in preclinical antibacterial R&D often do not disclose their preclinical pipeline until they are mature enough to approach the clinical stage.

Ongoing challenges within the preclinical antibacterial research landscape contributing to the high turnover rates also account for the limited technical expertise, scientific resources, and financial capacity available to drive progress in this area. These factors impact the future clinical pipeline of new antibacterial agents.

### From 2020, the composition of the pipeline by biological modality is relatively stable, with direct-acting small molecules, bacteriophage-based therapies, and indirect-acting small molecules being the most represented

**Fig 4:**
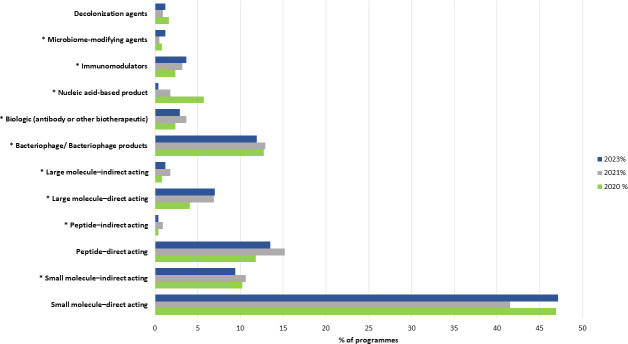
Distribution of preclinical pipeline projects by biological modality over the last three pipeline reviews. Note: data were available for this analysis from 2020.

Historically, antibacterial R&D focused on traditional bactericidal or bacteriostatic agents. By far, direct-acting small molecules (note that a small molecule is an organic compound with a molecular weight ≤1,000 Da; these molecules have generally a bactericidal or bacteriostatic effect) still represent the largest proportion of programs (about 50%). However, a variety of approaches are being pursued by researchers in the area, diverting their focus away from traditional agents (12, 17, 18). This shift is reflected by the increased exploration of non-traditional agents, as indicated by a diverse array of approaches emerging since the 2020 preclinical pipeline beyond the conventional small chemical molecules (Fig. 4). Among non-traditional agents, bacteriophage-based therapies and indirect-acting small molecule programs are prominently represented and account together for nearly 22% of the 2023 preclinical pipeline. These two categories of non-traditional agents present distinct characteristics and specific challenges for their development. Indirect-acting small molecules, which often target virulence determinants or inhibit exotoxins secreted by bacteria, are designed to complement or enhance the efficacy of standard-of-care treatments (usually direct-acting agents) rather than directly killing bacteria. Despite the encouraging focus in preclinical settings on non-traditional approaches, the way to patients is paved by several additional challenges. The clinical use of bacteriophages has primarily been limited to emergency use authorization applications to regulatory authorities. These applications allow for compassionate use on a case-by-case basis. Although there are ongoing randomized clinical trials employing bacteriophages, they are also anticipated to be administered as adjunctive therapies. Consequently, the development pathway for these add-on therapies, indirect-acting small molecules and bacteriophages (17, 19–22), often requires superiority clinical trial designs to demonstrate their added benefits as improved clinical outcomes when used in combination with existing treatments (23). Study design, along with quality and regulatory constraints, pose a challenge in their development and eventual approval for widespread clinical use.

Overall, despite the high turnover year-on-year of both unique research groups and research programs, the composition of the pipeline by biological modality has been relatively stable. This suggests that the surge in the number of non-traditional agents, initially observed in 2020 using this classification method, has now reached a point of stabilization (Fig. 4). This might be associated with the maturation or consolidation of non-traditional agents in preclinical development.

### The 2023 preclinical pipeline sees an increase in the number of programs reaching the final stage of preclinical development (the IND enabling phase) progressing to clinical trials compared to previous years

**Fig 5:**
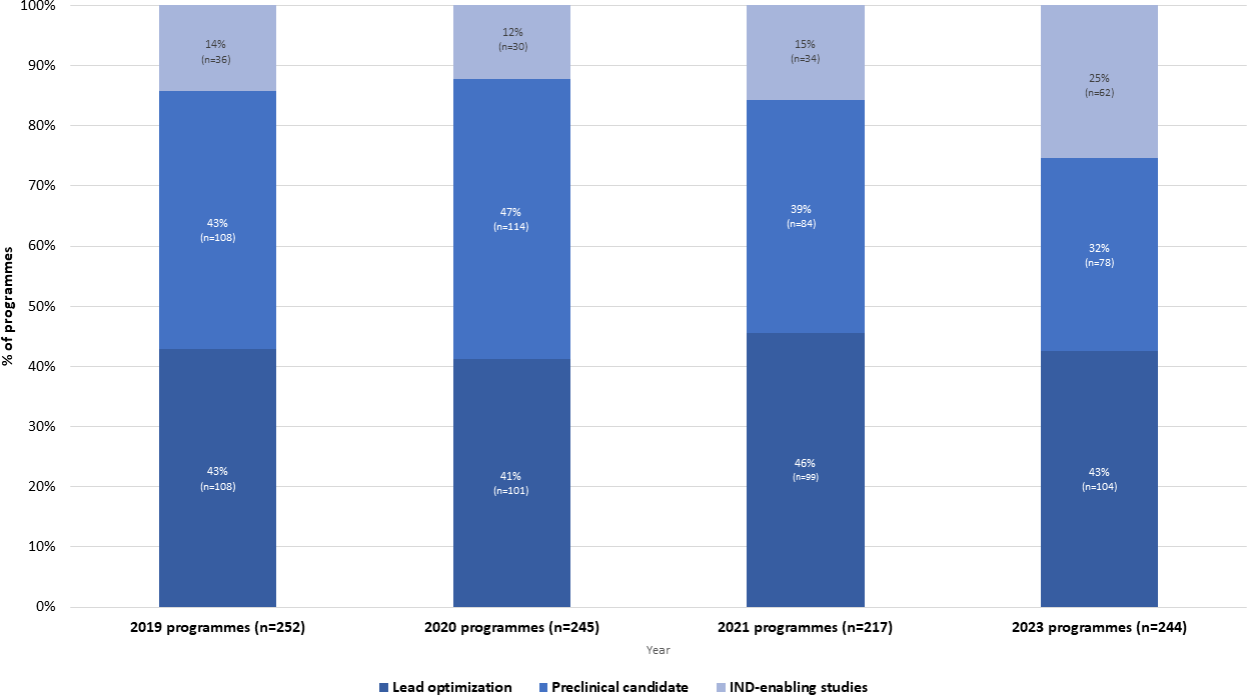
Categorization of programs by self-declared stage of preclinical development from 2019 to 2023.

Several analyses were performed to understand the progression and the dynamics of the preclinical ecosystem between 2019 and 2023. Research programs were grouped based on their self-declared preclinical development stage and compared to data collected in previous years (Fig. 5). The relative proportion of programs in each stage of development remained relatively stable over the 2019–2021 period. This suggests that as projects either fail or progress into clinical development, they are replaced by new programs entering the preclinical stages. However, in 2023, there was a significant increase in programs (*n* = 62, representing a 25.4% increase) that were in the IND-enabling phase of preclinical development as compared to earlier years.

A closer inspection of these programs in the IND-enabling phase in 2023 revealed that 19 of them had remained in the same stage of development since 2021. This is not surprising as pre-IND is one of the most critical stages where quality as well as toxicity issues usually arise. Meanwhile, 24 programs had transitioned into this later phase from earlier stages in 2021. Also, 19 new programs not mentioned in 2021 emerged in this later stage of development, contributing significantly to the growth observed. The notable growth in programs reaching the IND-enabling phase is encouraging but also raises questions about whether this will be sustained in the future or whether it may be due to the time gap since the last pipeline analysis.

### Most preclinical antibacterial research programs have a direct membrane effect according to the molecular mode of action

Preclinical programs were also categorized by molecular mode of action (Table 1). Antibacterial candidates with a “direct cell membrane effect” represented the largest proportion (28%) of research programs each year. This category encompasses both bacteriophage and bacteriophage-based endolysins, as well as the majority of the direct-acting peptide programs, highlighting the significance of strategies targeting bacterial cell membranes in the preclinical development landscape.

**TABLE 1:** Distribution of programs by mechanism of action, 2020–2023^*a*^

Mode-of-action category	2020 *n* (%)	2021 *n* (%)	2023 *n* (%)
Antivirulence	22 (9.0)	24 (11.1)	25 (10.2)
Cell wall synthesis—β-lactams and/or β-lactamase inhibitor	13 (5.3)	8 (3.7)	7 (2.9)
Cell wall synthesis—other	27 (11.0)	29 (13.4)	32 (13.1)
Central metabolism	6 (2.4)	7 (3.2)	10 (4.1)
Direct membrane effect	62 (25.3)	56 (25.8)	68 (27.9)
DNA replication/synthesis	16 (6.5)	12 (5.5)	16 (6.6)
Protein synthesis	28 (11.4)	18 (8.3)	18 (7.4)
RNA synthesis	5 (2.0)	3 (1.4)	5 (2.0)
Immunomodulation	9 (3.7)	10 (4.6)	14 (5.7)
Other cellular function	17 (6.9)	16 (7.4)	17 (7.0)
Potentiator or enabling agent	7 (2.9)	10 (4.6)	6 (2.5)
Not disclosed	19 (7.8)	9 (4.1)	14 (5.7)
Unknown	12 (4.9)	12 (5.5)	11 (4.5)
De-colonization	2 (0.8)	3 (1.4)	1 (0.4)
Total	245 (100)	217 (100)	244 (100)

^*a*^ In 2019, products were categorized differently which did not allow a direct comparison.

There is a renewed interest from many research groups in exploring the use of bacteriophage or phage-based therapies as a potential solution for addressing AMR. These endeavors embrace diverse strategies. Some groups concentrate on developing products capable of treating infections caused by specific bacterial species prevalent across many patients within a population subgroup. This approach aims to establish a generalized treatment option effective against particular bacterial strains, often achieved through the creation of phage cocktails.

Concurrently, other research groups are assembling collections of well-characterized bacteriophages, forming phage banks for patient-specific use in a personalized approach. This precision medicine strategy involves screening bacterial isolates from patients against phage libraries to determine susceptibility. Additionally, developers are establishing phage banks by screening wastewater or sewage water from specific regions, anticipating that this preparedness will enable a quicker response and shorter treatment duration. Despite the importance of this approach, when it was possible to distinguish among the different strategies, phage-based personalized treatments were excluded from the present analysis as they are intended for a single-patient use.

Some other groups are exploring genetic engineering of phages using various methods to enhance efficacy, modulate host range, or improve critical attributes such as biofilm penetration. For instance, engineered phage with antibacterial CRISPR-Cas have been shown to selectively reduce *Escherichia coli* in animal models (24). Synthetic biology methodologies are also employed to augment bacterial eradication by incorporating antimicrobial genes or proteins into designed phages (25). Advancements in machine learning are expected to streamline phage design processes, fostering greater efficiency in this area of research. The proportion of preclinical antibacterial agents by mode of action remained relatively stable between 2019 and 2023 (Table 1).

### Narrow-spectrum research programs represent more than one-third of the 2023 preclinical pipeline, a proportion that has remained roughly comparable across the years since the first WHO analysis in 2019

**Fig 6:**
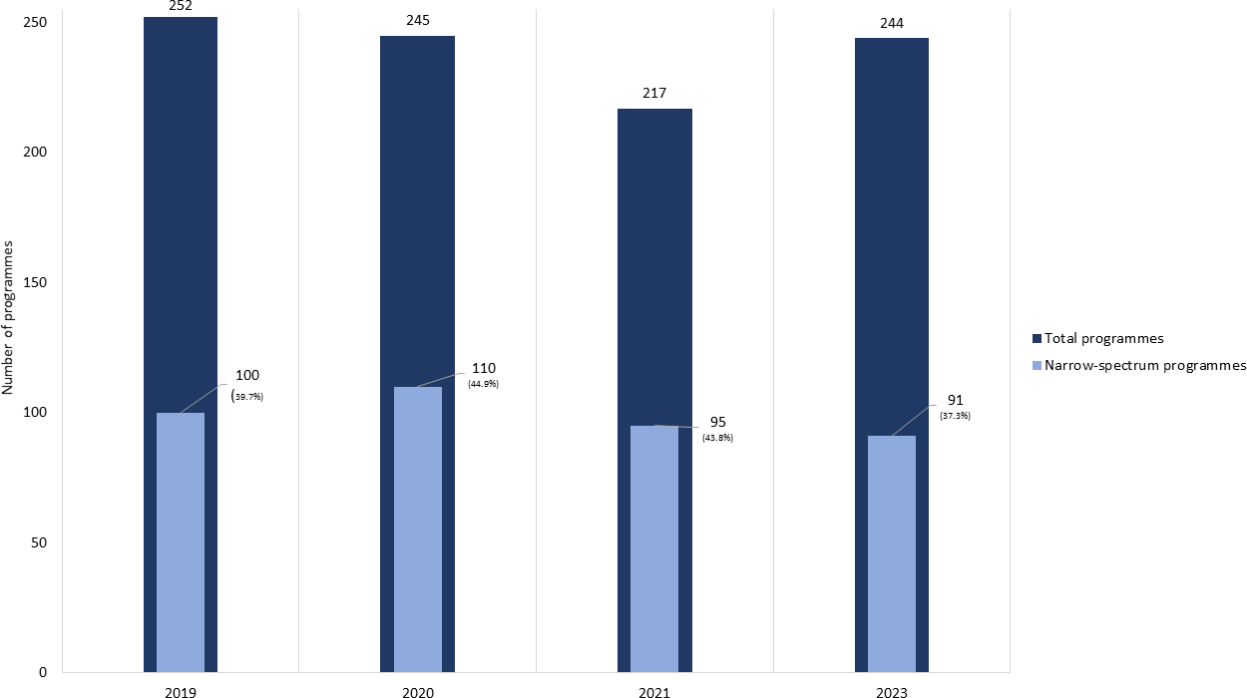
Analysis of narrow-spectrum programs across four consecutive preclinical pipeline reviews. Note: for the sole purpose of this analysis, narrow spectrum refers to agents that target a single species.

While many antibacterial agents typically have a broad spectrum of activity, the first preclinical pipeline analyses revealed quite a strong focus on species-specific programs targeting single WHO priority pathogens. The percentage of the antibacterial candidates in the pipeline that were pathogen specific ranged from a high of 44.9% in 2020 to a low of 37.3% in 2023 (Fig. 6).

**Fig 7:**
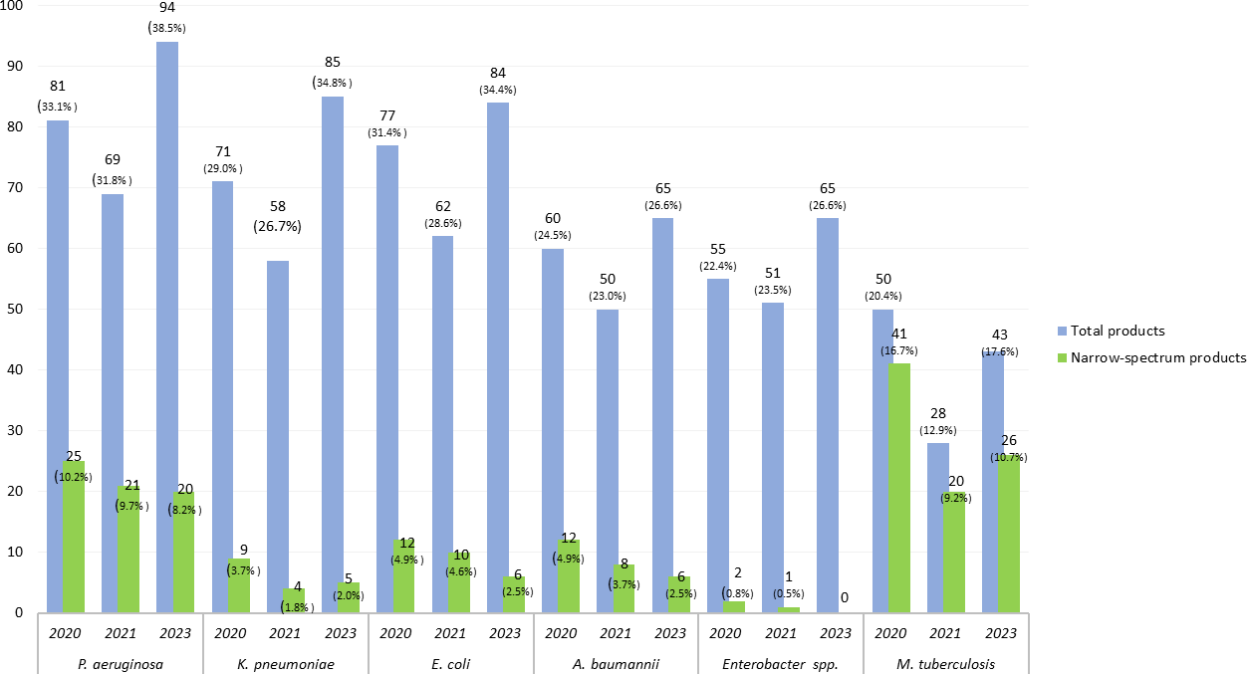
Analysis of narrow-spectrum programs by pathogen across four consecutive preclinical pipeline reviews.

Between 2019 and 2023, narrow-spectrum programs consistently targeted two pathogens: *Pseudomonas aeruginosa* (representing between 18% and 23% of species-specific programs, depending on the year) and *Mycobacterium tuberculosis* (representing a high of 43% of species-specific programs in 2019 and a low of 21.1% in 2021) (Fig. 7).

### The number of preclinical programs by intended target is fluctuating across the years of analysis

**Fig 8:**
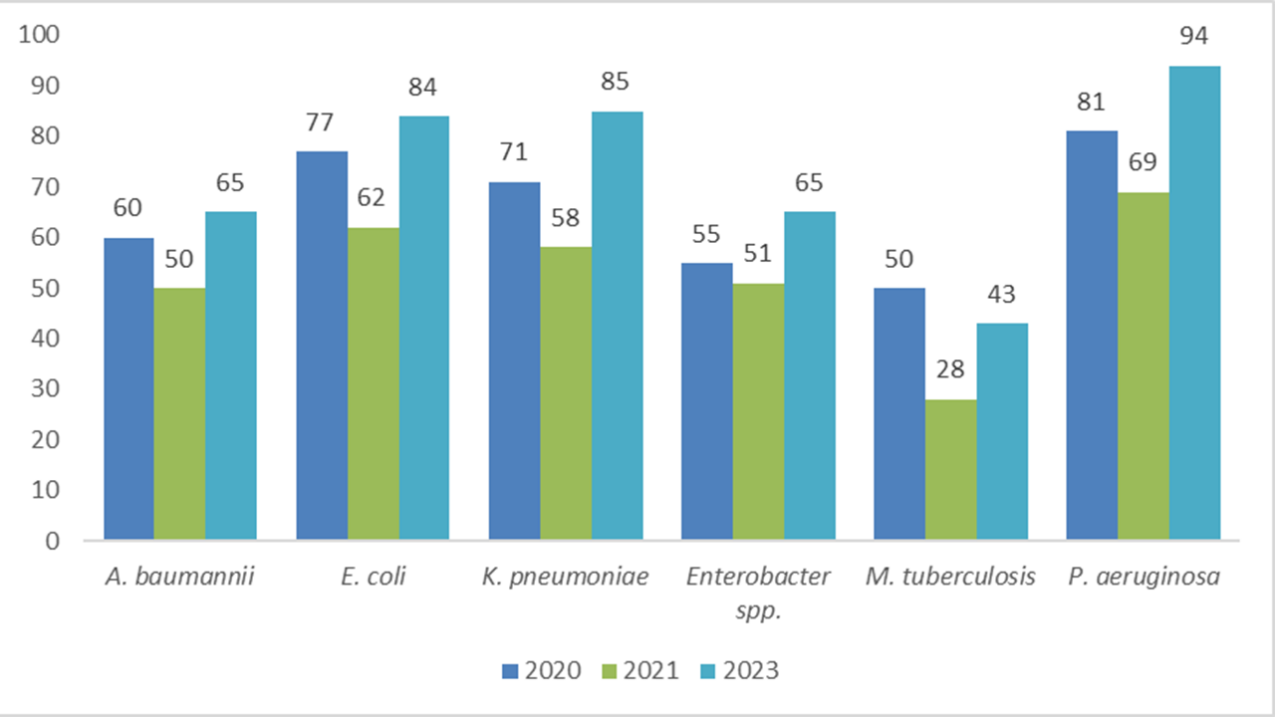
Analysis of programs by pathogen across three consecutive preclinical pipeline reviews.

With the introduction of the updated BPPL in 2024 (13), the clusters of priority pathogens have changed. However, a trend was explored for programs targeting the critical priorities across the last years of the analysis. The total number of products with declared activity against *Acinetobacter baumannii*, *E. coli*, *Klebsiella pneumoniae*, *Enterobacter* spp., and *M. tuberculosis* slightly declined from 2020 to 2021 to increase back in 2023 (Fig. 8).

This might be due to the stagnation in research due to coronavirus disease or to an underreporting of programs from researchers to the WHO data call. In 2019, data were collected in an aggregated format, thus precluding the analysis of the number of potential candidates against each bacterial priority pathogen.

Despite carbapenem-resistant *P. aeruginosa* being categorized as “high” in the 2024 BPPL due to a decrease in resistance, it is ranked sixth in terms of contribution to the global burden of infectious diseases (1), and thus, R&D efforts should be kept focused on this pathogen. In the 2023 preclinical pipeline, there is significant effort to develop both broad- and narrow-spectrum agents against this pathogen.

When looking at agents in the medium group of the BPPL, there appears to be a comparatively limited focus on agents targeting *Streptococcus pneumoniae*, which ranks fourth for the burden of mortality (1), although this may be impacted by the large focus on preventative immunization strategies for this species.

Certain limitations were noted when performing this review. The WHO annual preclinical data call was performed against the 2017 BPPL, while the finalization of the 2024 BPPL, which incorporates several changes compared to the previous one, was ongoing. To mitigate the potential reporting bias sponsors were requested to also submit information against difficult-to-treat bacteria not included in the 2017 BPPL. However, some programs may not have been submitted to the call, potentially leading to underrepresentation of programs targeting these species in the pipeline analysis. In addition, many developers may not have yet evaluated the activity of their candidates against a full panel of bacterial species due to the early development stage and therefore may not have known and reported potential activity against some of the pathogens.

## CONCLUSION

The WHO has now completed four global analyses of the publicly available preclinical antibacterial pipeline projects since 2019, providing a valuable snapshot of the activity in the research ecosystem. Through these longitudinal analyses, several trends have become apparent, shedding light on critical aspects of the early-stage antibacterial R&D. The overall number of products remains constant over the 4 years of analysis. Since the start of the observation in 2019, the number of traditional small molecules and non-traditional agents has found a steady footing within the preclinical antibacterial R&D landscape.

The preclinical antibacterial pipeline predominantly relies on microsized (<10 employees) and small-sized (<50 employees) entities and academic institutions to progress the early discovery science and development of innovative treatment products for drug-resistant infections. While there is a broad geographical distribution of preclinical pipeline projects, these are heavily focused toward Europe and the United States of America. This is strictly linked and fully aligns with preexisting infrastructure and expertise, regulatory and scientific know-how, national or regional strategies, and funding—mostly from government or non-profit foundations—available in these regions. At the same time, there is a clear need to prioritize a more equitable and global approach to promote antibacterial R&D worldwide as R&D groups based in regions of higher burden and unmet need would be especially motivated to focus on local concerns. That said, achieving registration of a compelling new therapeutic candidate will necessarily entail a global development program that anticipates the need to ensure global and equitable access after marketing authorization. This shall focus on the fragile populations that are the most exposed to drug-resistant infections and that would benefit the most from accessing innovative antibacterial agents. Regardless of their location worldwide, the small numbers of both antibacterial programs and the research groups/companies (and their rates of attrition) make it imperative to mobilize more support and funding for early discovery research as well as translational work to progress programs into viable clinical candidates.

While the preclinical pipeline showcases a large variety of product types, the primary focus remains on Gram-negative pathogens. Antibiotics have been traditionally broad-spectrum agents, although in recent years, we have seen an increased interest in narrow-spectrum agents that could potentially mitigate AMR if coupled with appropriate diagnosis. However, the shift toward narrow-spectrum agents focusing on a single pathogen appears to have now plateaued. The development of species-specific agents will likely require increased use of rapid diagnostics to ensure adequate enrichment of the enrolled patient population with the pathogen of interest. Post-approval, effective, and affordable diagnostics with a quick turnaround will be key to ensuring appropriate use, preventing their relegation to second-line therapy or add-on to combination therapies.

Overall, the preclinical pipeline remains innovative and dynamic with many non-traditional approaches potentially necessitating innovative clinical trial designs (20). This is especially true for products that are likely to be used in combination with standard-of-care drugs. Consequently, clinical benefit will need to be demonstrated in a randomized controlled trial. When reviewing the later stages of preclinical candidates in development, it is evident that while some programs advance, many others fail to make meaningful progress.

Multiple factors, including scientific and technical challenges, high turnover rates, a prevalence of a paucity of small privately funded entities and a notable absence of larger pharmaceutical companies, access to sustainable funding, and competing public health priorities could have contributed to the observed lack of progression. These factors once again highlight the general fragility of the preclinical antibacterial ecosystem. As a global community, it is our collective responsibility to devise better solutions to ensure the development and the sustainability of a diverse pipeline of antibacterial agents capable of delivering enough innovative products to meet the growing demands of patients in need.

## References

[1] Collaborators AR. 2022. Global burden of bacterial antimicrobial resistance in 2019: a systematic analysis. Lancet 399:629–655. doi:10.1016/S0140-6736(21)02724-0 35065702 PMC8841637

[2] The World Bank. 2017. Drug-resistant infections: a threat to our economic future. The World Bank/Topics/Health, Geneva, Switzerland. Available from: https://www.worldbank.org/en/topic/health/publication/drug-resistant-infections-a-threat-to-our-economic-future. Retrieved 26 Mar 2024.

[3] OECD Publishing. 2023. Embracing a one health framework to fight antimicrobial resistance. OECD Health Policy Studies, Paris, France. Available from: 10.1787/ce44c755-en. Retrieved 26 Mar 2024.

[4] World Health Organization. 2019. Ten threats to global health in 2019. WHO/Health topics, Geneva, Switzerland. Available from: https://www.who.int/news-room/spotlight/tenthreats-to-global-health-in-2019. Retrieved 26 Mar 2024.

[5] World health organization. 2024. In 2023 Antibacterial agents in clinical and preclinical developmentand. Geneva, Switzerland.

[6] Outterson K, Orubu ESF, Rex J, Årdal C, Zaman MH. 2022. Patient access in 14 high-income countries to new antibacterials approved by the US food and drug administration, European medicines agency, Japanese pharmaceuticals and medical devices agency, or health Canada, 2010–2020. Clin Infect Dis 74:1183–1190. doi:10.1093/cid/ciab612 34251436 PMC8994582

[7] O’Neill J, Wellcome Foundation and UK Department of Health. 2014. Antimicrobial resistance: tackling a crisis for the health and wealth of nations. Available from: https://amr-review.org/sites/default/files/160525_Final%20paper_with%20cover.pdf. Retrieved 26 Mar 2024.

[8] Tacconelli E, Carrara E, Savoldi A, Harbarth S, Mendelson M, Monnet DL, Pulcini C, Kahlmeter G, Kluytmans J, Carmeli Y, Ouellette M, Outterson K, Patel J, Cavaleri M, Cox EM, Houchens CR, Grayson ML, Hansen P, Singh N, Theuretzbacher U, Magrini N, WHO Pathogens Priority List Working Group. 2018. Discovery, research, and development of new antibiotics: the WHO priority list of antibiotic-resistant bacteria and tuberculosis. Lancet Infect Dis 18:318–327. doi:10.1016/S1473-3099(17)30753-3 29276051

[9] World Health Organization. 2017. Prioritization of pathogens to guide discovery, research and development of new antibiotics for drug-resistant bacterial infections, including tuberculosis. Available from: https://iris.who.int/handle/10665/311820. Retrieved 26 Mar 2024.

[10] Blasco B, Jang S, Terauchi H, Kobayashi N, Suzuki S, Akao Y, Ochida A, Morishita N, Takagi T, Nagamiya H, et al.. 2024. High-throughput screening of small-molecules libraries identified antibacterials against clinically relevant multidrug-resistant A. baumannii and K. pneumoniae. eBioMedicine 102:105073. doi:10.1016/j.ebiom.2024.105073 38520916 PMC10963893

[11] Rex J, Bradford P. Really, Really Hard. 48,015 → 0: Antibacterial Discovery Is Hard. Really, Really Hard. Available from: https://amr.solutions/2024/04/09/48015-0-antibacterial-discovery-is-hard-really-really-hard/. Accessed 4 April 2024

[12] World Health Organization. 2019. Antibacterial agents in preclinical development: an open access database. Geneva, Switzerland

[13] World Health Organization. 2024. WHO Bacterial Priority Pathogens List, 2024: bacterial pathogens of public health importance to guide research, development and strategies to prevent and control antimicrobial resistance.. Geneva, Switzerland

[14] Wellcome Trust. 2020. It’s time to fix the antibiotic market. Available from: https://wellcome.org/news/its-time-fix-antibiotic-market. Retrieved 26 Mar 2024.

[15] AMR Industry Alliance. 2024. Leaving the lab: tracking the decline in AMR R&D professionals. Available from: https://www.amrindustryalliance.org/wp-content/uploads/2023/02/Leaving-the-Lab_final-1.pdf. Retrieved 26 Mar 2024.

[16] Sun D, Gao W, Hu H, Zhou S. 2022. Why 90% of clinical drug development fails and how to improve it? Acta Pharm Sin B 12:3049–3062. doi:10.1016/j.apsb.2022.02.002 35865092 PMC9293739

[17] Czaplewski L, Bax R, Clokie M, Dawson M, Fairhead H, Fischetti VA, Foster S, Gilmore BF, Hancock REW, Harper D, Henderson IR, Hilpert K, Jones BV, Kadioglu A, Knowles D, Ólafsdóttir S, Payne D, Projan S, Shaunak S, Silverman J, Thomas CM, Trust TJ, Warn P, Rex JH. 2016. Alternatives to antibiotics – a pipeline portfolio review. Lancet Infect Dis 16:239–251. doi:10.1016/S1473-3099(15)00466-1 26795692

[18] Konwar AN, Hazarika SN, Bharadwaj P, Thakur D. 2022. Emerging non-traditional approaches to combat antibiotic resistance. Curr Microbiol 79:330. doi:10.1007/s00284-022-03029-7 36155858 PMC9510247

[19] UK Parliament. 2024. The antimicrobial potential of Bacteriophages. First Report of Session 2023–24. London, United Kingdom . https://publications.parliament.uk/pa/cm5804/cmselect/cmsctech/328/report.html.

[20] Uyttebroek S, Chen B, Onsea J, Ruythooren F, Debaveye Y, Devolder D, Spriet I, Depypere M, Wagemans J, Lavigne R, Pirnay J-P, Merabishvili M, De Munter P, Peetermans WE, Dupont L, Van Gerven L, Metsemakers W-J. 2022. Safety and efficacy of phage therapy in difficult-to-treat infectios: ans: a systematic review. Lancet Infect Dis 22:e208–e220. doi:10.1016/S1473-3099(21)00612-5 35248167

[21] Gibb B, Hyman P, Schneider CL. 2021. The many applications of engineered bacteriophages-an overview. Pharmaceuticals (Basel) 14:634. doi:10.3390/ph14070634 34208847 PMC8308837

[22] McCallin S, Drulis-Kawa Z, Ferry T, Pirnay J-P, Nir-Paz R, ESGNTA – ESCMID study group for non-traditional antibacterials. 2023. Phages and phage-borne enzymes as new antibacterial agents. Clin Microbiol Infect:S1198-743X(23)00528-1. doi:10.1016/j.cmi.2023.10.018 37866680

[23] Rex JH, Fernandez Lynch H, Cohen IG, Darrow JJ, Outterson K. 2019. Designing development programs for non-traditional antibacterial agents. Nat Commun 10:3416. doi:10.1038/s41467-019-11303-9 31366924 PMC6668399

[24] Gencay YE, Jasinskytė D, Robert C, Semsey S, Martínez V, Petersen AØ, Brunner K, de Santiago Torio A, Salazar A, Turcu IC, et al.. 2024. Engineered phage with antibacterial CRISPR-Cas selectively reduce E. coli burden in mice. Nat Biotechnol 42:265–274. doi:10.1038/s41587-023-01759-y 37142704 PMC10869271

[25] Lenneman BR, Fernbach J, Loessner MJ, Lu TK, Kilcher S. 2021. Enhancing phage therapy through synthetic biology and genome engineering. Curr Opin Biotechnol 68:151–159. doi:10.1016/j.copbio.2020.11.003 33310655 PMC11996084

